# Deep learning system assisted detection and localization of lumbar spondylolisthesis

**DOI:** 10.3389/fbioe.2023.1194009

**Published:** 2023-07-19

**Authors:** Jiayao Zhang, Heng Lin, Honglin Wang, Mingdi Xue, Ying Fang, Songxiang Liu, Tongtong Huo, Hong Zhou, Jiaming Yang, Yi Xie, Mao Xie, Liangli Cheng, Lin Lu, Pengran Liu, Zhewei Ye

**Affiliations:** ^1^ Department of Orthopedics, Union Hospital, Tongji Medical College, Huazhong University of Science and Technology, Wuhan, China; ^2^ Intelligent Medical Laboratory, Union Hospital, Tongji Medical College, Huazhong University of Science and Technology, Wuhan, China; ^3^ Department of Orthopedics, Nanzhang People’s Hospital, Nanzhang, China; ^4^ Department of Orthopedics, Daye People’s Hospital, Daye, China; ^5^ Department of Orthopedics, Renmin Hospital of Wuhan University, Wuhan, China

**Keywords:** artificial intelligence, deep learning, lumbar spondylolisthesis, diagnosis, assisted diagnosis

## Abstract

**Objective:** Explore a new deep learning (DL) object detection algorithm for clinical auxiliary diagnosis of lumbar spondylolisthesis and compare it with doctors’ evaluation to verify the effectiveness and feasibility of the DL algorithm in the diagnosis of lumbar spondylolisthesis.

**Methods:** Lumbar lateral radiographs of 1,596 patients with lumbar spondylolisthesis from three medical institutions were collected, and senior orthopedic surgeons and radiologists jointly diagnosed and marked them to establish a database. These radiographs were randomly divided into a training set (*n* = 1,117), a validation set (*n* = 240), and a test set (*n* = 239) in a ratio of 0.7 : 0.15: 0.15. We trained two DL models for automatic detection of spondylolisthesis and evaluated their diagnostic performance by PR curves, areas under the curve, precision, recall, F1-score. Then we chose the model with better performance and compared its results with professionals’ evaluation.

**Results:** A total of 1,780 annotations were marked for training (1,242), validation (263), and test (275). The Faster Region-based Convolutional Neural Network (R-CNN) showed better precision (0.935), recall (0.935), and F1-score (0.935) in the detection of spondylolisthesis, which outperformed the doctor group with precision (0.927), recall (0.892), f1-score (0.910). In addition, with the assistance of the DL model, the precision of the doctor group increased by 4.8%, the recall by 8.2%, the F1-score by 6.4%, and the average diagnosis time per plain X-ray was shortened by 7.139 s.

**Conclusion:** The DL detection algorithm is an effective method for clinical diagnosis of lumbar spondylolisthesis. It can be used as an assistant expert to improve the accuracy of lumbar spondylolisthesis diagnosis and reduce the clinical workloads.

## Introduction

Spondylolisthesis implies forward translation of the superior vertebra relative to the adjacent inferior vertebra on the sagittal plane (Hu et al., 2008). The etiology of spondylolisthesis has not been well known. A large number of studies have shown that congenital developmental defects and chronic strain or stress injury are possibly the two main causes, especially the latter (Jones and Rao, 2009). The primary pathological features of spondylolisthesis are the destruction of the anatomical structure of the lumbar spine and the accompanying pressure on nerves, causing clinical symptoms such as pain, numbness, and bladder/bowel dysfunction (Tumialan, 2019). In the early stages of the disease, most patients with lumbar spondylolisthesis have none obvious clinical symptoms (Guigui and Ferrero, 2017).

According to relevant guidelines, the lumbar lateral radiograph in a standing position is the most appropriate non-invasive examination method for detecting spondylolisthesis (Matz et al., 2016). Some relevant studies have shown that variability between inter-observer and intra-observer in lumbar spine diagnosis can be as high as 15% (Butt and Saifuddin, 2005). Also, this variation may increase if there involves a rotation. Considering that the progression of the disease may severely reduce the patients’ quality of life and increase social and medical burdens (Karsy and Bisson, 2019), early and accurate diagnosis of lumbar spondylolisthesis is critical.

Among various forms of artificial intelligence (AI), machine learning is the most widely applied (Le, 2022; Sunnetci and Alkan, 2023). As an emerging research direction in the field of machine learning, deep learning (DL) has been widely used in medical image analysis, medical decision support systems, protein function prediction, genomics research and medical natural language processing (Le and Huynh, 2019; Zhou et al., 2022; Foersch et al., 2023; Lee et al., 2023; Theodoris et al., 2023). Especially in the field of medical image analysis, there are relevant studies using deep learning algorithms for identification and diagnosis of diseases such as lung nodules, retinal lesions, ovarian cancer, skin diseases and so on (Gao et al., 2022; Kim et al., 2022; Ruamviboonsuk et al., 2022; Zhang et al., 2022). In orthopedics, some scholars have also carried out related researches on using DL technology to identify limb and spine fractures, and intervertebral disc herniation lesions through X-ray, CT, MRI and other image data to demonstrate the advantages of AI in this field (Li et al., 2021; Liu et al., 2022; Zheng et al., 2022). Therefore, AI-assisted lumbar spondylolisthesis detection may become an effective auxiliary diagnosis method. If the performance of the AI model is stable and convincing, it can be used to locate the spondylolisthesis in the lateral X-ray film, thereby assisting orthopedists or radiologists in diagnosing spondylolisthesis to improve diagnostic accuracy.

In the study, we proposed a DL algorithm to diagnose spondylolisthesis using only lumbar lateral radiographs. 1) To train the best model using the multi-center dataset and ensure stable detection performance; 2) To compare diagnostic performance between the detection model and clinical doctors; 3) To compare the clinician’s diagnostic performance with and without the assistance of the DL model. It was expected that our DL model could act as an assistant to help doctors reduce medical errors during diseases detection and treatment.

## Methods

### Patients

This is a retrospective study involving multiple institutions. The study was approved by the Institutional Ethics Committee, and the requirement for informed consent was waived due to the retrospective nature of the study and negligible risks involved. Patients whose conditions fit for the study were selected from the databases of the Union Hospital Affiliated to Tongji Medical College of Huazhong University of Science and Technology, Daye People’s Hospital, and Nanzhang County People’s Hospital.

**FIGURE 1:**
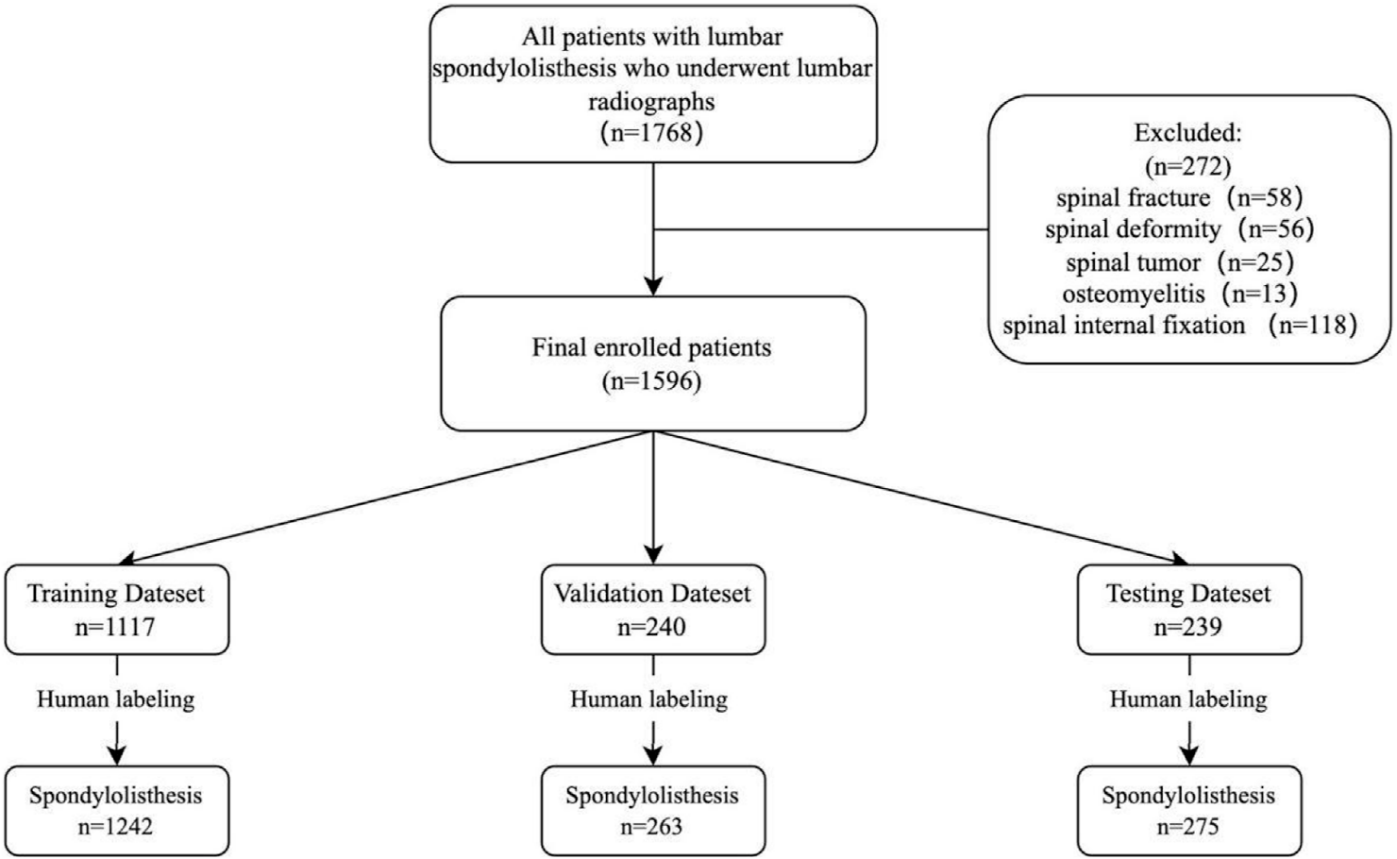
Research flow chart. The graph shows the sample numbers in training, validation, and test datasets. All datasets (*n* = 1,596 patients) were randomly divided into training (*n* = 1,117), validation (*n* = 240) and testing (*n* = 239) sets in a ratio of 0.7: 0.15: 0.15.

In this retrospective study, the inclusion criteria were as follows: 1) patients aged 18 years or older; 2) patients diagnosed with lumbar spondylolisthesis; 3) including complete lateral imaging of the lumbar spine. The criteria for exclusion included: 1) patients with a history of spinal fracture, deformity, tumor, osteomyelitis, or internal fixation; 2) poor X-ray imaging; 3) foreign objects obstructing affect image reading. Multiple spondylolisthesis observed from plain radiographs was not an exclusion criterion. Finally, a total of 1,596 radiographs from patients with spondylolisthesis diagnosed between December 2017 to December 2022 were selected for this study. The detailed selection process is shown in Figure 1.

### Diagnosis

The lateral X-ray images were analyzed and graded by a chief orthopedic physician with more than 15 years of experience in spine specialties and a chief physician of imaging department with more than 15 years of professional experience in imaging. In cases of disagreement, a chief orthopedic expert with 20 years of clinical experience reviewed the patient’s clinical information and additional imaging examination, and arrived at a conclusion after discussions with those two physicians.

**FIGURE 2:**
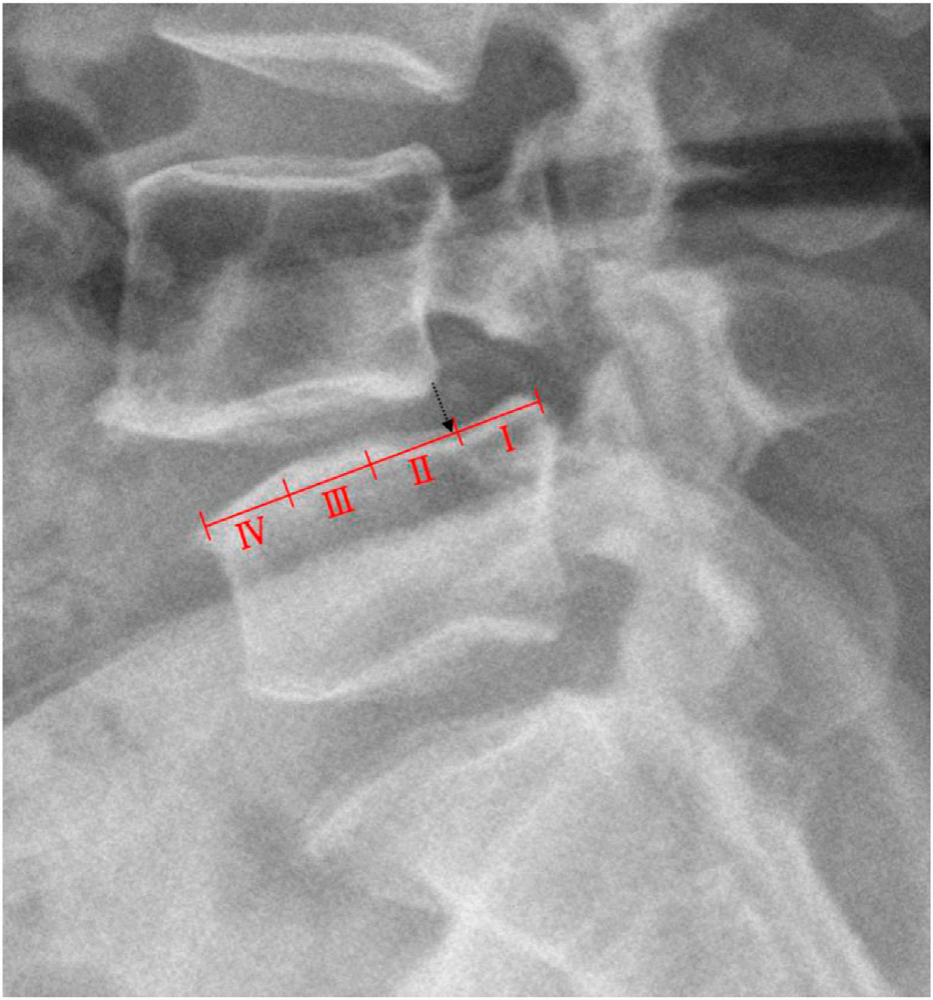
Schematic diagram of the Meyerding classification measuring method.

The classification of lumbar spondylolisthesis is divided into 4 grades according to the Meyerding classification method (Tumialan, 2019). The anterior-posterior diameter (AP) of the upper surface of the lower vertebral body is divided into four equal parts, degree I is within 1/4, degree II is between 1/4 and 2/4, degree III is between 2/4 and 3/4, while over 3/4 is graded IV (Figure 2).

### Imaging annotation

All the acquired X-ray images of the lumbar spine were stored in high-quality JPEG format. And the region of interest (RoI) of each image was manually annotated by experienced orthopedic surgeons and radiologists based on the previous image diagnosis results. The RoIs of the posterior vertebral body were manually labeled using the Labelme software package (https://github.com/wkentaro/labelme).

The diagnosis of lumbar spondylolisthesis is mainly based on the position relationship between the upper and lower vertebral bodies. Therefore, this study will focus on the posterior edge area of lumbar vertebrae in X-rays, and train algorithms by annotating the posterior edge areas of normal and slipped vertebrae to accurately identify the location of slippage as much as possible.

### Data sets for training, validation, and testing

We selected 1,596 lateral lumbar spine radiographs (one plain radiograph per person) taken from December 2017 to December 2022 for training, validation and testing (Table 1). These radiographs were randomly distributed in a ratio of 0.7: 0.15: 0.15 using the Python program, with 1,117 distributed for training, 240 for validation, and 239 for testing. 1,242 spondylolisthesis were identified in the training set, 263 spondylolisthesis in the validation set, and 275 spondylolisthesis in the testing set (Figure 1).

**TABLE 1:** Demographics of patients were included.

	Training	Vaidation	Testing	Control date Set	*p*-value
	Data Set	Data Set	Data Set		
No.of patients	1,117 (60.87)	240 (13.08)	239 (13.02)	239 (13.02)	-
Age	58 (51–66)	60 (52–67)	59 (51–67)	59 (51–67)	0.724
Male	324 (29.01)	60 (25.00)	67 (28.15)	64 (26.78)	0.776
No. of annotations	1,242 (69.78)	263 (14.78)	275 (15.45)	-	-

Data are presented as n (%) or median (IQR).

### Data set processing

Data was processed to ensure the detection accuracy of the target. The imported images were resized with the shorter edge’s dimensions fixed and pixels to 800, and the longer side was resized proportionally. At the same time, there is a 50% probability for each image to be flipped horizontally, increasing the richness of the image. In addition, through image normalization, the value of features (or images) were adjusted to a similar range, which speeds up network convergence. Specifically, image normalization is the process of transforming a raw image to a unique standardized form through a series of transformations in order to eliminate the influence of other transformation functions on the image. In this study, we used common normalization parameters: mean = [123.675, 116.28, 103.53] and std = [58.395, 57.12, 57.375]. These parameters can make different images comparable and improve model training effectiveness.

### DL model architecture and implementation

**FIGURE 3:**
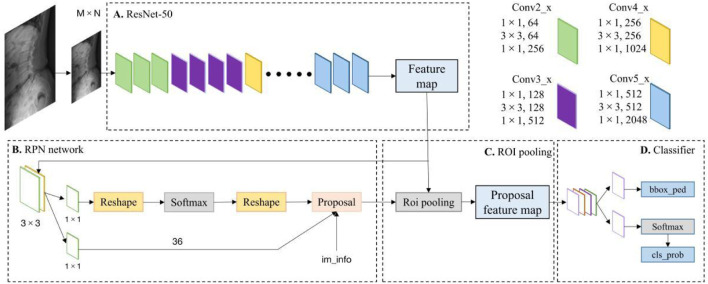
Faster R-CNN network structure for lumbar spondylolisthesis detection.

**FIGURE 4:**
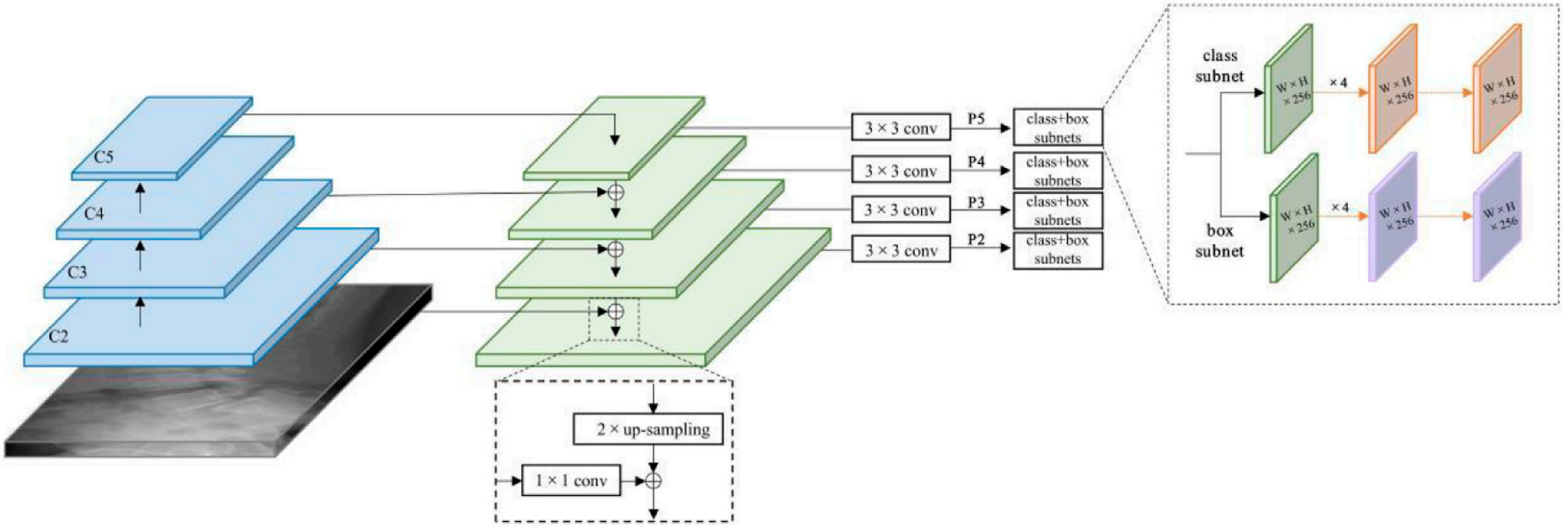
RetinaNet network structure for lumbar spondylolisthesis detection.

Two DL detection models, Faster R-CNN (Ren et al., 2017) and RetinaNet (Lin et al., 2017), were selected for research. Faster R-CNN is a typical algorithm in the two-stage object detection model, which mainly includes four parts: Conv Layers, Region Proposal Networks (RPN), RoI Pooling and Classifier (Figure 3). Faster RCNN has integrated feature extraction, a region proposal, bounding box regression (rect refine), and classification into one network, which greatly improves the overall performance, especially in terms of detection speed. RetinaNet is a one-stage high-accuracy deep learning algorithm that uses a Feature Pyramid Network (FPN) chelation on CNN as its backbone network. It attaches two sub-networks respectively for anchor box regression and classification for each level to achieve accurate recognition of location information and object detection (Figure 4).

We ran the DL framework-pytorch on the Ubuntu 16.04 operation system (http://www.ubuntu.com) with an NVIDIA V100 GPU (CUDA 10.2 and cuDNN 7.6.5) (http://developer.nvidia.com), 32 GB VRAM.

### Non-maximum suppression (NMS)

The above model might predict many overlapping bounding boxes, and we used NMS to select the best one from multiple overlapping predicted bounding boxes. NMS is a post-processing method. For multiple overlapping detection boxes, NMS sorts all the detection results according to the score from high to low, keeps the box with the highest score, and deletes the rest.

### Model comparison and validation

In order to objectively evaluate the classification performance of Faster R-CNN and Retina, images in the testing set were used to evaluate the classification accuracy of the two models after training. By drawing the PR curve and calculating the AP value and the three evaluation indicators (precision, recall rate and F1-score), we obtained the performance difference of two models in classification. These indicators were presented in detail in the Supplementary Material.

Furthermore, to validate the performance of the DL models in images without spondylolisthesis, lateral lumbar radiographs of 239 subjects (diagnosed with lumbar muscle strain or intervertebral disc herniation) without spondylolisthesis were added as a control group, and the number of false positives (FPs) in the control group was calculated.

### Comparison of results of DL models and professional physicians

To compare the difference in diagnosing spondylolisthesis by DL algorithms and doctors, we set up a group of six doctors, and compared their results with those of the DL model. The six doctors in this group are all orthopedic surgeons with more than 5 years of orthopedic experience and passed the intermediate certificate examination (none of the six doctors were involved in diagnosis or labeling). We recorded the precision, sensitivity, F1-score, and the diagnosis time of the doctor group. Four weeks later, the diagnostic test of the doctor group assisted by AI was carried out, in which the images were shuffled.

Since each lateral lumbar radiograph may have multiple locations of lumbar spondylolisthesis while generally more parts are normal, receiver operating characteristic (ROC) curves and specificities are not applicable here. Setio et al. (2017) show that free-reaction ROC (fROC) allows multiple lesions and normal sites to appear on one image, so we used a fROC curve to analyze the sensitivity and average FPs of the CNN model to the independent test set, and the curve covered the points of six non-AI-assisted physician diagnosis results and six AI-assisted physician diagnosis results.

### Compare the detection effect of images with different resolutions

To study the impact of resolution on detection of lumbar spondylolisthesis, we used images with different resolutions for detection and plotted corresponding fROC curves.

### Statistical analysis

Continuous variables were presented as median [interquartile range (IQR)]. Categorical variables were expressed by counts and percentages. For the comparison of baseline characteristics among different data sets, the ANOVA test was used for continuous variables and χ2 test was used for categorical variables. Precision, recall, F1-score, TP, FN, and FP were selected as diagnostic performances, and the corresponding 95% confidence intervals were estimated using bootstrapping with 1,000 bootstraps. The paired-samples *t*-test was used to compare diagnostic performances of Faster R-CNN and RetinaNet. The Mann-Whitney was used to compare diagnostic performances of doctors with or without AI assistance. The Student’s t-test was used to compare diagnostic performances of Faster R-CNN and doctors without AI assistance. The bootstrapping were performed using packages “boot” of R 4.1.2 (The R Foundation for Statistical Computing, Vienna, Austria). Other statistical analyses were performed using SAS Statistics software (version 9.4, SAS Institute Inc., Cary, North Carolina, USA). All statistical tests were two-sided, and *p* < 0.05 was considered statistically significant.

## Results

### Demographic data of the included patients

There were no significant differences in age or sex between the training, validation, testing data sets and the control group (all *p* > 0.05) (Table 1).

### Comparison of the DL models

**FIGURE 5:**
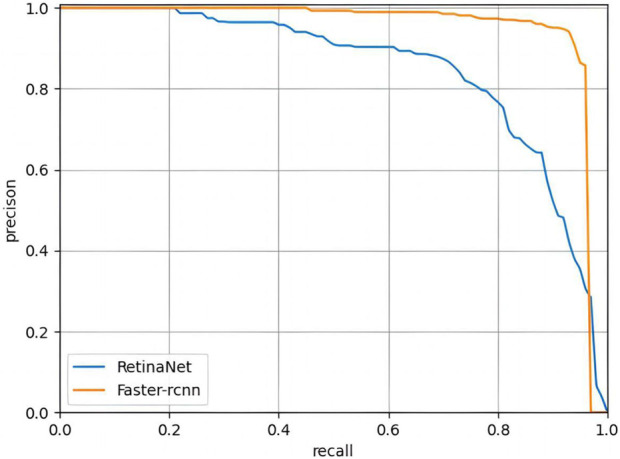
PR curves of Faster RCNN and RetinaNet. The PR curve of Faster R-CNN can completely wrap that of RetinaNet, indicating that Faster R-CNN is better than RetinaNet.

The PR curves output by the two trained DL models are shown in Figure 5, with AP of 0.966 and 0.902 for Faster R-CNN and Retina, respectively. In terms of classification performance, Faster R-CNN outperformed Retina in precision (0.935 > 0.794), Recall (0.935 > 0.771) and F1-Score (0.935 > 0.782). In the control group, FP was unavoidable, with FPs of 14 and 47 for the two models, respectively. Detailed results are provided in the Supplementary Material. Therefore, FasterR R-CNN was selected as the better model for further research.

### Clinical application of the DL model

**FIGURE 6:**
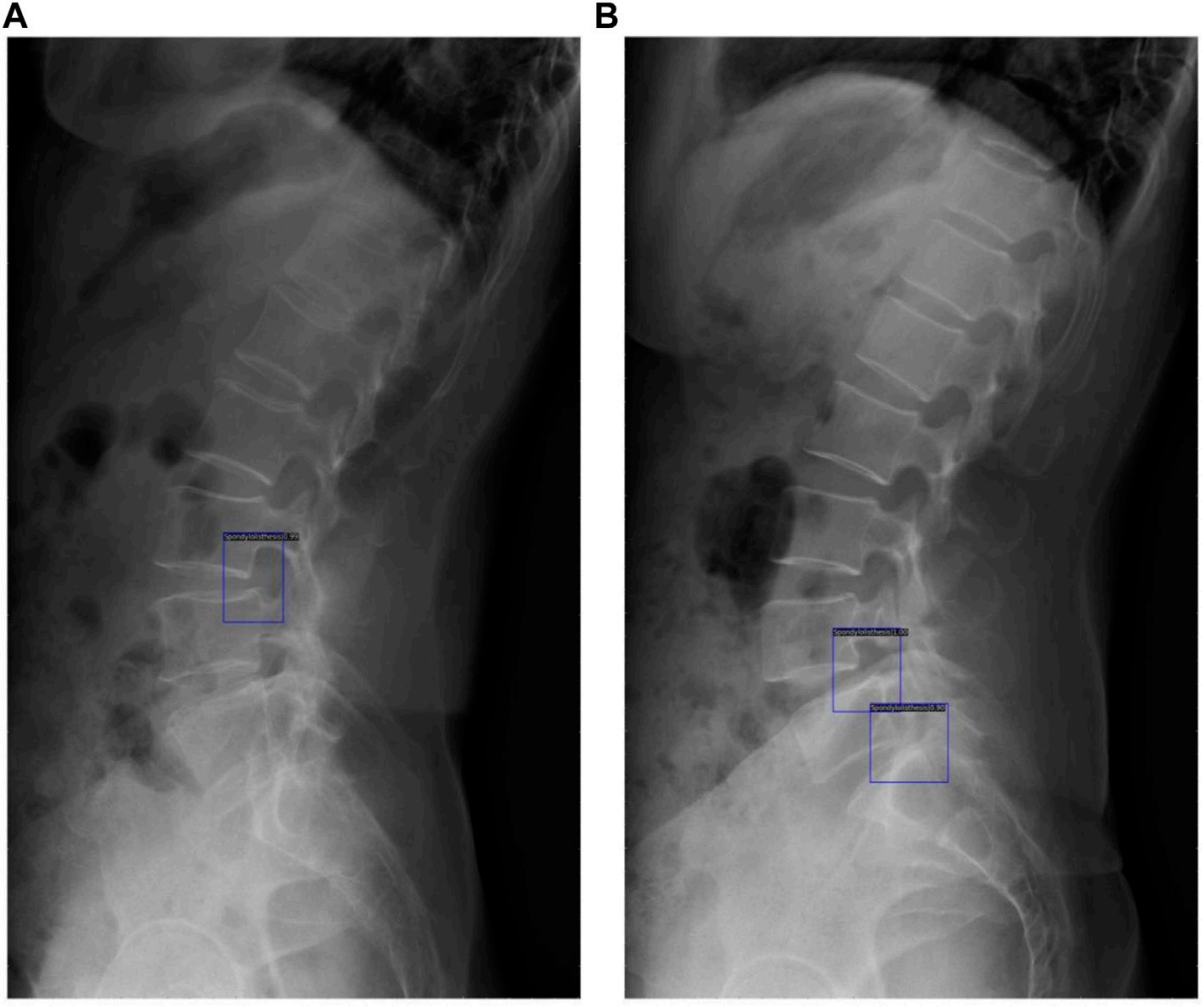
These results demonstrated the potential of clinical application of the DL model, with blue rectangles marking the location of spondylolisthesis and numbers showing the probability of spondylolisthesis **(A)** shows detection of one spondylolisthesis and **(B)** shows detection of two spondylolisthesis.

The original image was imported into the AI model and the model automatically executed the entire procedure and produced results with position markers and spondylolisthesis probability of spondylolisthesis (Figure 6). The time from importing a plain film to generating final results took approximately 0.167 s/piece on average, while the average time the doctors group took was about 25.452 s/piece under the same testing data set.

### Comparison of diagnostic performance of DL model and doctor group

**FIGURE 7:**
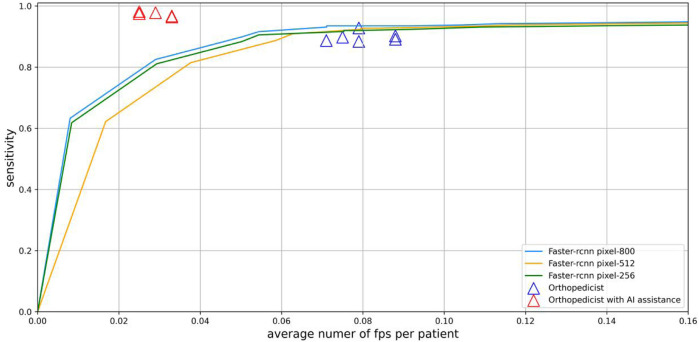
Sensitivity and average number of FPs per patient of spondylolisthesis on whole X-Ray images are shown by fROC curves. Physicians without AI assistance (purple triangles) are near the curve, and physicians with AI assistance (red triangles) are above and to the left of the curve.

The six points without AI assistance in the doctor group were all near the bottom of the fROC curve, and the six points with higher values represented diagnosis after AI assistance (Figure 7). The doctor group had an average precision of 0.927, sensitivity of 0.892, and F1-score of 0.910 in diagnosing spondylolisthesis. However, DL models performed slightly better in precision and significantly better in sensitivity and F1-Score (Table 2).

**TABLE 2:** Comparison of the DL and doctors.

	Faster R-CNN	Doctors without AI	*p*-value
Precision	0.935	0.927	0.126
Sensitivity	0.935	0.892	< 0.001
F1-score	0.935	0.910	< 0.001

As shown in Table 3. After AI assistance, the average precision of spondylolisthesis diagnosis in the doctor group increased by 4.8% from 0.927 to 0.975 (*p* = 0.004), the average sensitivity of diagnosis increased by 8.2% from 0.892 to 0.974 (*p* = 0.004), and the average F1 score increased by 6.4% from 0.910 to 0.974. Without AI assistance and with AI assistance, the average diagnosis time of the doctor group was 25.452 s and 18.355 s, respectively (*p* = 0.004), and the average diagnosis time of each plain X-ray film was shortened by 7.139 s with AI assistance.

**TABLE 3:** Comparison of the doctors with or without AI.

	Doctors without AI	Doctors with AI	*p*-value
Precision	0.927	0.975	0.004
Sensitivity	0.892	0.974	0.004
F1-score	0.910	0.974	0.004
Average Time	25.452	18.313	0.004

### The result of images with different resolutions

The results showed that when the resolution was reduced to 512 pixels, the detection performance decreased slightly, while a significant decrease in detection performance occurred when the resolution was reduced to 256 pixels (Figure 7).

## Discussion

In our study, we proposed a DL-based identification and diagnosis method for lumbar spondylolisthesis. The trained DL model, in the independent testing set, the precision of DL model for lumbar spondylolisthesis diagnosis was 0.935, recall was 0.935, and F1-score was 0.935. Compared with evaluation of attending physicians in orthopedics, the performance of the DL model was significantly higher. With the assistance of AI, the diagnostic performance of the doctor group has been significantly improved.

Lumbar spondylolisthesis is one of the most common spinal disorders, with studies showing that approximately 4%–6% of the population suffer from spondylolisthesis and lumbar spondylolisthesis (Fredrickson et al., 1984). Although there might be some mild symptoms of lumbar spondylolisthesis, in most cases, people usually do not feel it in early stages, which will lead to the further development of the disease (Wang et al., 2017). In addition, busy clinical work may cause doctors to make misdiagnosis and missed diagnosis, especially for patients with slippage less than 25%, which will seriously affect the diagnosis and treatment of lumbar spondylolisthesis (Iguchi et al., 2002). Therefore, an efficient and accurate automatic diagnosis system for spondylolisthesis is very important for early diagnosis, treatment and rehabilitation of the disease.

MRI/CT/X-rays can be used to detect spondylolisthesis, but according to the latest guidelines, lateral lumbar radiographs are the most appropriate non-invasive examination for spondylolisthesis, especially in the absence of reliable evidence. MRI is mostly used to evaluate the neurological status of spondylolisthesis patients with spinal stenosis, and CT mostly for patients with contraindications for MRI examination.

Early studies on AI diagnosis of spondylolisthesis primarily concentrated on CT/MRI images, which also produced positive outcomes. Liao et al. (2016) proposed an automated lumbar spondylolisthesis assessment method for automatic measurement of spondylolisthesis on CT images, and the method reached the level of radiologists performing annotation. Yunliang et al. (2017) developed an automatic detection system for spondylolisthesis based on supervised learning, which had a sensitivity of 91.8% and a specificity of 90.0% for spondylolisthesis detection on CT/MRI images. Zhao et al. (2019) proposed a FAR network for vertebral body detection and lumbar spondylolisthesis classification based on MRI images, with an accuracy of 0.8933 ± 0.0276. In terms of detection of X-ray images, Giam et al. (Trinh et al., 2022) developed LumbarNet for the detection and segmentation of vertebral bodies, and realized the judgment of lumbar spondylolisthesis, with an accuracy rate of 88.83%.

This study focused on the detection of spondylolisthesis in lumbar spine radiographs. We first proposed to detect spondylolisthesis by identifying the structure between the posterior borders of adjacent vertebral bodies, so as to achieve high-precision detection of spondylolisthesis on lateral lumbar radiographs. In this study, we developed and investigated two different types of algorithms for spondylolisthesis detection, where Faster R-CNN showed better performance than RetinaNet in spondylolisthesis detection. Faster R-CNN is a two-stage algorithm with real-time performance and higher detection accuracy, while RetinaNet is a state-of-the-art one-stage algorithm that focuses on detection speed (Sunnetci et al., 2023; Xu et al., 2023). Considering the high accuracy requirements by clinical work, Faster R-CNN is more suitable for the detection of spondylolisthesis.

Currently, mainstream target detection algorithms based on DL are mainly divided into two-stage and single-stage. The first stage of the two-stage algorithm works to identify candidate regions in the target image, and the subsequent stage to classify the candidate regions. Typical two-stage algorithms include Region-based Convolutional Neural Network (R-CNN), Fast R-CNN and Faster R-CNN, *etc.* Single-stage detection algorithms do not include the stage of candidate region proposal generation, and directly give class probabilities and spatial coordinates of objects. You only look once (YOLO) and RetinaNet are typical single-stage algorithms.

Analysis of the diagnostic results of the physician group showed that they frequently missed multiple or minor spondylolisthesis. However, Faster R-CNN extracts feature maps for each input image through region proposal networks and sliding window M × N feature maps, which leads to the algorithm being able to accurately detect spondylolisthesis (Karako et al., 2021). And the model can detect many mild spondylolisthesis that clinicians miss. In addition, the diagnosis time after AI assistance is significantly shortened. As we analyzed the network stage by stage, we found that the proposals obtained by the first-stage RPN network of Faster R-CNN were basically around the targets, which greatly reduced the number of false positives in the backgrounds. This further verifies the effectiveness of the first-stage network training. When these proposals are sent into the second-stage network, they can be regarded as weak priors and further refined in the second stage network. We found that our algorithm was able to eliminate close false positives in the second stage and further facilitate the performance of our network.

For the detection of images with different resolutions, when the resolution is reduced to 512, the detection effect slightly decreases, and when further reduced to 256, the detection effect significantly decreases. This may be in lateral lumbar spine images, the structure between vertebrae is a small target relative to the entire image. If the image resolution is lowered, information may be lost, making it difficult to fully reflect vertebral features and leading to a decrease in detection performance.

In detection, missed diagnosis is inevitable. When analyzing the images of missed diagnosis, we found that severe lumbar spondylolisthesis, especially those with the grade greater than 4, are prone to be missed. This may be because most patients only have mild to moderate lumbar spondylolisthesis, and the number of patients with severe lumbar spondylolisthesis is relatively small. During the training process, algorithms may not be able to fully learn about particularly severe lumbar spondylolisthesis. However, in actual clinical work, this type of severe lumbar spondylolisthesis is rarely missed because its degree of slippage is severe and obvious on plain films. This suggests that we need to collect more data to improve algorithm performance.

This study still has some limitations. First, the DL algorithm is designed to detect lumbar spondylolisthesis, but since it would be affected by the shape of the vertebral body, especially the shape of the posterior edge of the vertebral body, cases with a medical history of fractures, tumors, osteomyelitis, and internal fixation operations are excluded. Therefore, users should consider this major limitation. Second, this model can only identify whether there is spondylolisthesis on the lateral lumbar spine radiograph, but the type of spondylolisthesis (true spondylolisthesis, pseudospondylolisthesis, or degenerative spondylolisthesis) still needs to be comprehensively analyzed by the doctor based on the patient’s medical history and other image details and data. Third, the basic diagnostic data was mainly analyzed and assessed by an orthopedist and a radiologist who specialized in the spine. The limited number of specialists involved may compromise the reliability of the DL models’ performance. If more experienced doctors participate in diagnosis and labeling, human bias can be effectively reduced, thereby improving research validity. Finally, although we have used data from three hospitals and the amount of data is large enough, and the training effect is good, it is still not enough for a mature and excellent algorithm. Therefore, in the future, we will collect more data from hospitals and establish larger multi-center databases and external validation sets to further improve algorithm performance.

Although these factors indicate that there is room for further investigation, we believe that the rigor and standards we demonstrated for the research ensured its value and the overall results deserve full consideration. If the performance of the algorithms we proposed can be validated by others, this fast and accurate model has great potential to assist doctors in emergency rooms, and outpatient and inpatient clinics. Besides, in areas with limited resources and a shortage of medical professionals, this model could provide spondylolisthesis prediagnosis from readily available X-ray images.

## Conclusion

We developed a DL model to diagnose and locate lumbar intervertebral position of spondylolisthesis. Compared with the orthopedic surgeons, the DL model showed significant advantages in the diagnostic accuracy and speed of lumbar spondylolisthesis. The results proved that our approach is feasible and demonstrated the excellent performance of the DL model in the diagnosis of lumbar spondylolisthesis. This technology is expected to become a second expert, assisting clinicians to improve the accuracy of lumbar spondylolisthesis diagnosis and properly reduce the frontline doctors’ workloads.

## Data Availability

The original contributions presented in the study are included in the article/Supplementary Material, further inquiries can be directed to the corresponding authors.
