# Supplementary material for: Deep learning system assisted detection and localization of lumbar spondylolisthesis
Source: Front Bioeng Biotechnol. 2023 Jul 19;11:1194009. doi: 10.3389/fbioe.2023.1194009 (PMC10394621; doi:10.3389/fbioe.2023.1194009)
Supplement: Supplementary file 1 [file DataSheet1.DOCX]

**SUPPLEMENTARY MATERIAL**

## **Detailed Explanation of Evaluation Indicators**

The research on spondylolisthesis detection involves binary classification. We use precision, recall, F1 score, PR curve, and average precision (AP) to evaluate the performance of the model.

Precision is evaluated from the perspective of prediction results, that is, how many of the data judged as positive samples are true positive samples. It is calculated as follows:

$$Precision=\frac{\mathrm{TP}}{TP+FP}$$

Recall and Sensitivity are the same concept and are evaluated from the real sample set, that is, how many positive samples were retrieved out of the total positive samples. It is calculated as follows:

$$Recall/S\mathrm{ensitivity}=\frac{\mathrm{TP}}{TP+FN}$$

The F1 score is an indicator of classification problems and is considered to be as important as recall and precision. It is the harmonic mean of precision and recall, with a maximum of 1 and a minimum of 0. It is calculated as follows:

$$F1=2\times\frac{Precision\times Recall}{Precision+Recall}$$

The PR curve is the curve of precision and recall. In the PR curve, the ordinate is precision and the abscissa is recall. The PR curve reflects the trade-off between the classifier’ recognition accuracy of positive examples and the coverage ability of positive examples.

Average Precision (AP) is the average of the precision at different recall points, and it is shown as the area under the PR curve. The larger the value of AP, the higher the average precision of the model.

TP, FP and FN in the above equation are defined as:

FP (False Positive): judged as a positive sample but actually a negative sample.

FN (False Negative): judged as a negative sample but actually a positive sample.

TP (True Positive): judged as a positive sample and actually a positive sample.

## **Detailed Performance of Two Detection Models**

The detailed performance of Faster R-CNN and RetinaNet such as precision, recall, and F1-score is shown in **Supplementary Table 1**.

**Supplementary Table 1. Comparison of Faster R-CNN and RetinaNet**

| Metrics | Faster R-CNN | RetinaNet | *p value* |
| --- | --- | --- | --- |
| TPs | 257 (244, 269) | 212 (193, 226) | <0.001 |
| FNs | 18 (10, 28) | 63 (49, 78) | <0.001 |
| FPs | 18 (11, 26) | 55 (39, 71) | <0.001 |
| Precision | 257/275 = 0.935  (0.904, 0.959) | 212/267 = 0.794  (0.740, 0.839) | <0.001 |
| Recall | 257/275 = 0.935  (0.897, 0.960) | 212/275 = 0.771  (0.717, 0.818) | <0.001 |
| F1-score | 1.748/1.870 = 0.935  (0.913, 0.952) | 1.224/1.565 = 0.782  (0.741, 0.817) | <0.001 |
| Control Set FPs | 14  (7, 21) | 47  (33, 61) | <0.001 |

Using bootstrapping with 1000 bootstraps to estimate the corresponding 95% confidence intervals, which is shown inside parentheses

## **Detailed Results of the Doctor Group Test**

**Supplementary Table 2** shows the detailed results of six physicians’ diagnosis without AI assistance, and **Supplementary Table 3** shows the six physicians diagnosis with AI assistance.

**Supplementary Table 2. Diagnostic performance of 6 orthopedicists**

| Indicators | Orthopedicists | | | | | |
| --- | --- | --- | --- | --- | --- | --- |
|  | 1 | 2 | 3 | 4 | 5 | 6 |
| TPs | 248  (233, 260) | 245  (228, 258) | 244  (226, 258) | 247  (232, 261) | 245  (228, 258) | 243  (227, 258) |
| FNs | 27  (17, 37) | 30  (20, 40) | 31  (21, 41) | 28  (19, 39) | 30  (20, 39) | 32  (22, 42) |
| FPs | 21  (13, 30) | 19  (11, 28) | 17  (9, 25) | 18  (10, 26) | 21  (13, 30) | 19  (11, 27) |
| Precision | 248/269 = 0.922  (0.888, 0.950) | 245/264 = 0.928  (0.895, 0.954) | 244/261 = 0.934  (0.903, 0.961) | 247/265 = 0.932  (0.900, 0.957) | 245/266 = 0.921  (0.889,0.947) | 243/262 = 0.927  (0.894, 0.954) |
| Sensitivity | 248/275 = 0.902  (0.862, 0.932) | 245/275 = 0.891  (0.850, 0.922) | 244/275 = 0.887  (0.839, 0.921 ) | 247/275 = 0.898  (0.861, 0.931) | 245/275 = 0.891  (0.848, 0.923) | 243/275 = 0.884  (0.844, 0.920) |
| F1-score | 1.663/1.824 = 0.912  (0.885, 0.934) | 1.654/1.819 = 0.909  (0.882, 0.93 ) | 1.659/1.822 = 0.910  (0.877, 0.930) | 1.674/1.830 = 0.915   (0.887, 0.937) | 1.641/1.812 = 0.906  (0.878, 0.928) | 1.639/1.811 = 0.905  (0.876, 0.928) |
| Average time (s) | 25.752 | 27.345 | 28.013 | 23.671 | 24.371 | 23.561 |

Using bootstrapping with 1000 bootstraps to estimate the corresponding 95% confidence intervals, which is shown inside parentheses

**Supplementary Table 3. Diagnostic performance of 6 orthopedicists with AI assistance**

| Indicators | Orthopedicists | | | | | |
| --- | --- | --- | --- | --- | --- | --- |
|  | 1 | 2 | 3 | 4 | 5 | 6 |
| TPs | 266  (254, 278) | 268  (256, 281) | 270  (259, 282) | 265  (250, 276) | 268  (257, 280) | 269  (257, 281) |
| FNs | 9  (4, 15) | 7  (2, 13) | 5  (1, 9) | 10  (4, 16) | 7  (2, 12) | 6  (2, 11) |
| FPs | 8  (3, 14) | 6  (2, 11) | 6  (2, 11) | 8  (3,14) | 6  (2, 11) | 7  (2, 12) |
| Precision | 266/274 = 0.971 (0.944, 0.985) | 268/274 = 0.978  (0.955, 0.989) | 270/276 = 0.978  (0.955,0.992) | 265/273 = 0.971  (0.948, 0.985) | 268/274 = 0.978   (0.956, 0.989) | 269/276 = 0.975  (0.948, 0.989) |
| Sensitivity | 266/275 = 0.967  (0.942, 0.985) | 268/275 = 0.975  (0.950, 0.989) | 270/275 = 0.982  (0.957, 0.993) | 265/275 = 0.964  (0.937, 0.982) | 268/275 = 0.975  (0.949, 0.989) | 269/275 = 0.978 (0.953, 0.990) |
| F1-score | 1.878/1.938 = 0.969  (0.953, 0.981) | 1.907/1.943 = 0.976  (0.960, 0.987) | 1.921/1.960 = 0.98  (0.966, 0.989) | 1.872/1.935 = 0.967  (0.949, 0.981) | 1.907/1.953 = 0.976  (0.962, 0.987) | 1.907/1.953 = 0.976   (0.961, 0.987) |
| Average time (s) | 18.873 | 18.563 | 19.733 | 16.354 | 17.651 | 18.954 |

Using bootstrapping with 1000 bootstraps to estimate the corresponding 95% confidence intervals, which is shown inside parentheses
